# On the current role of hydratases in biocatalysis

**DOI:** 10.1007/s00253-018-9065-7

**Published:** 2018-05-21

**Authors:** Matthias Engleder, Harald Pichler

**Affiliations:** 1 Austrian Centre of Industrial Biotechnology (acib), Petersgasse 14, 8010 Graz, Austria; 2 Institute of Molecular Biotechnology, Graz University of Technology, NAWI Graz, BioTechMed Graz, Petersgasse 8010 Graz, Austria

**Keywords:** Hydro-lyase, Hydratase, Oleate hydratase, Kievitone hydratase, Linalool dehydratase-isomerase

## Abstract

Water addition to carbon-carbon double bonds provides access to value-added products from inexpensive organic feedstock. This interesting but relatively little-studied reaction is catalysed by hydratases in a highly regio- and enantiospecific fashion with excellent atom economy. Considering that asymmetric hydration of (non-activated) carbon-carbon double bonds is virtually impossible with current organic chemistry, enzymatic hydration reactions are highly attractive for industrial applications. Hydratases have been known for several decades but their biocatalytic potential has only been explored over the past 15 years. As a result, a considerable amount of information on this enzyme group has become available, enabling their development for practical applications. This review focuses on hydratases catalysing water addition to non-activated carbon-carbon double bonds, and examines hydratases from a biochemical, structural and mechanistic angle. Current challenges and opportunities in hydration biocatalysis are discussed, and, ultimately, their potential for organic synthesis is highlighted.

## Introduction

Hydratases (EC 4.2.1.x) catalyse the selective addition of water to carbon-carbon double bonds, and thereby generate primary, secondary or tertiary alcohols from prochiral substrates (Hanefeld and Resch 2015). Since they allow for hydration of alkenes with up to 100% atom efficiency, hydratases are of huge interest for the synthesis of chiral building blocks and products for chemical industries. Hydratases are generally well expressed in commonly used recombinant hosts, show good activity for physiological substrates, and their reactions do not require cofactor recycling. Consequently, they are viable alternatives to other enzyme systems currently examined or already used for biocatalytic hydroxyl functionalisations, such as mono-oxygenases, lipoxygenases or epoxide hydrolases (Roper and Grogan 2015; Hiseni et al. 2015).

From a mechanistic point of view, hydration reactions are divided into two classes depending on the substrate properties (Chen et al. 2015; Resch and Hanefeld 2015). Water addition to an electron-deficient, activated carbon-carbon double bond in α,β-unsaturated carbonyls is performed via nucleophilic Michael addition (Tokoroyama 2010). Water addition to electron-rich, isolated carbon-carbon double bonds is an electrophilic addition following the rule of Markovnikov (Kerber 2002). Despite the bad reactivity of water, both classes are common natural reactions, which renders enzymatic hydration an even more compelling type of biotransformation (van der Werf et al. 1994; Anderson et al. 2006). In contrast, asymmetric transformations with synthetic hydration catalysts are strongly opposed by the poor nucleophilicity and electrophilicity of water, and are often associated with harsh reaction conditions, formation of adverse side products and/or complete lack of selectivity (Resch and Hanefeld 2015). This leads to the infrequent use of acid- or base-catalysed chemical hydrations in organic synthesis (Resch and Hanefeld 2015) and to the few reports on selective hydration routes by means of chemical synthesis (Xue et al. 2004; Wang et al. 2007; Boersma et al. 2010). Hydratases are able to cope with these inherent restraints by virtue of providing a carefully orchestrated environment for chiral (bio)synthesis, i.e. by elaborate active site arrangement or the supply of nucleotide and metal cofactors. In fact, since asymmetric chemical hydration is impossible with state-of-the-art techniques, the selective addition of water to non-activated carbon-carbon double bonds was recently highlighted as an organic chemist’s ‘dream reaction’ (Schnapperelle et al. 2012; Gröger 2014).

One of the major persisting limitations of many hydratases results from their eminent role in primary metabolism. Especially the Michael addition is part of many core metabolic pathways, with prominent examples being reactions of amino acid synthesis (e.g. dehydroquinate dehydratase) or the citric acid cycle (aconitase and fumarase). While the high substrate specificity of hydratases involved in these reactions is of utmost importance for cellular functionality, it contradicts the demand set for an enzyme catalyst in industrial biotransformations, which should show broad substrate tolerance (Faber 2011; Nestl et al. 2014; Payer et al. 2017). An exception is the production of (*S*)-malic acid on a 2500 t a^−1^ scale with a fumarase as one of the few examples for successful implementation of a naturally occurring hydratase reaction into an industrial process (Liese et al. 2006).

**Fig. 1:**
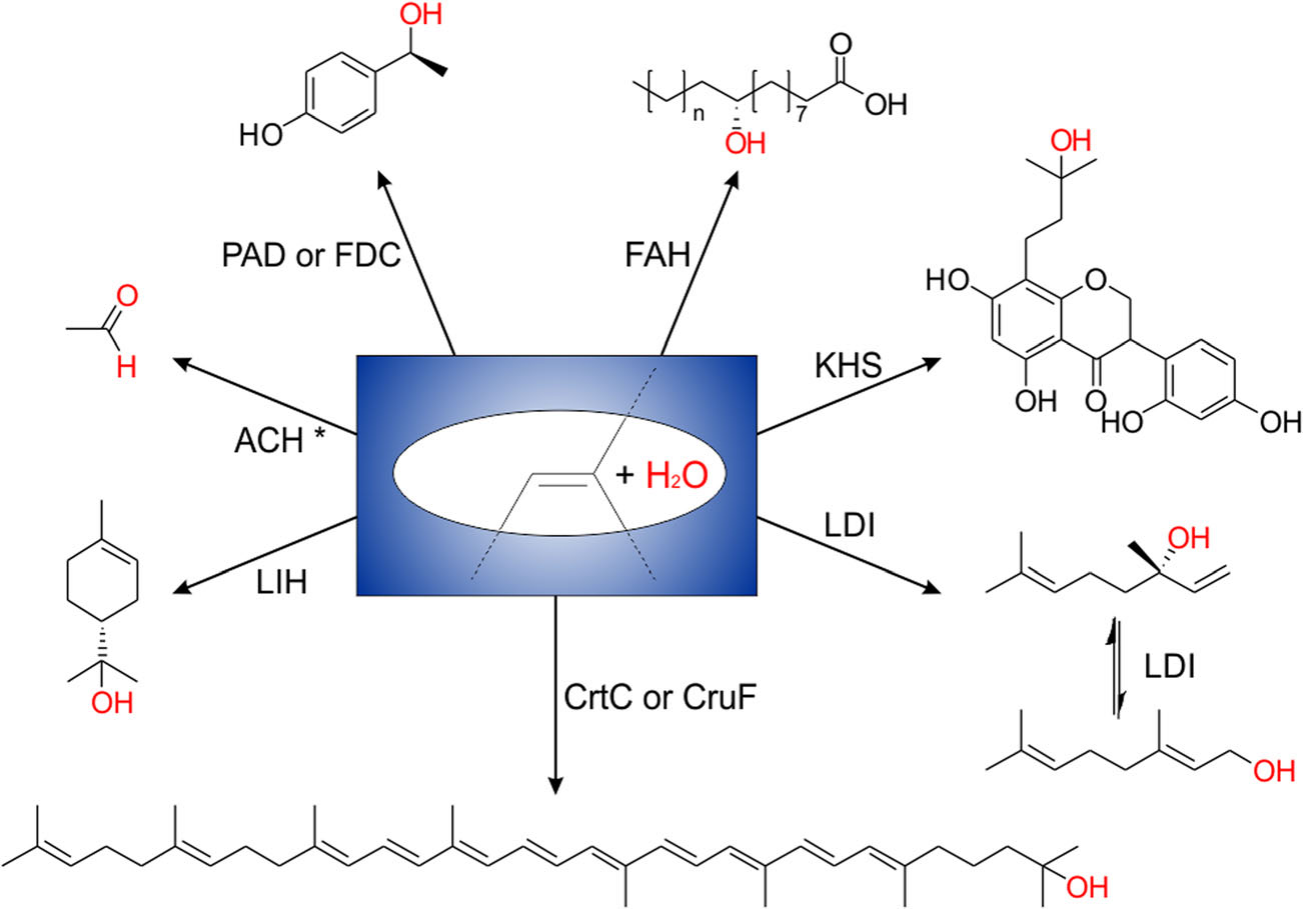
Typical water addition reactions to non-activated carbon-carbon double bonds catalysed by hydratases. FAH = fatty acid hydratase; KHS = kievitone hydratase; LDI = linalool dehydratase-isomerase; CrtC and CruF = carotenoid-1,2-hydratases; LIH = limonene hydratase; ACH = acetylene hydratase; PAD = phenolic acid decarboxylase; FDC = ferulic acid decarboxylase. The asterisk in the ACH catalysed hydration of acetylene indicates hydration of a carbon-carbon triple bond

Discovery of and research on hydratases for hydroxyl-functionalisation of non-activated carbon-carbon double bonds has accelerated remarkably over the past years. The quantity of information on this enzyme class is increasing steadily and their enormous potential for industrial biocatalysis is being unveiled. Here, we focus on the relevant enzyme group catalysing water addition to non-activated carbon-carbon double bonds in view of their biochemical, structural, and mechanistic properties (Fig. 1). We discuss current drawbacks and future challenges to be met and suggest perspectives that will allow for a broad application of hydratases in near future.

## Enzymes for hydration and their properties

### Fatty acid hydratases

The enzymatic addition of water to free fatty acids is catalysed by fatty acid hydratases (FAHs). Although no strict convention for classification of FAHs has been implemented, they are mostly referred to as oleate hydratases (EC 4.2.1.53) due to their high activity for hydration of oleic acid (OA). With more than 25 characterized representatives, FAHs are the most broadly studied group of enzymes hydrating non-activated carbon-carbon double bonds. Already when the enzymatic production of a hydroxy fatty acid was first reported (Wallen et al. 1962), speculations on fatty acid alcohol formation by addition of water were emerging (Niehaus and Schroepfer 1965). The hydration mechanism was, however, confirmed only substantially later by bioconversions of OA to hydroxy fatty acids in medium enriched either with D_2_O or H_2_^18^O (Koritala et al. 1989).

Since the discovery of FAHs, fatty acid alcohol formation was shown in many different organisms (Davis et al. 1969; el-Sharkawy et al. 1992; Hudson et al. 1995, 1998; Kaneshiro et al. 1995; Kishimoto et al. 2003), but it was not until 2009 that an enzyme catalysing hydration of OA to 10-hydroxystearic acid (10-HSA) was described and isolated from *Elizabethkingia meningoseptica* (OhyA), the strain originally known as *Pseudomonas* sp. 3266 and described for hydroxy fatty acid production in 1962 (Bevers et al. 2009).

Despite notable advances in the identification of new FAHs in recent years, only little is known on their actual physiological function in microorganisms. The few studies focusing thereon reported either an effect on the metabolisation of potentially toxic unsaturated fatty acids (Marounek et al. 2003; Zheng et al. 2005; Volkov et al. 2010; Connerth et al. 2010; Ortega-Anaya and Hernández-Santoyo 2015), assigned a role in stress protection (Rosberg-Cody et al. 2011) or concluded an impact on host-microbe interaction due to alteration of the cell hydrophobicity (Chen et al. 2016). Despite this lack of physiological knowledge, plenty of biochemical and structural data on FAHs is arising. All currently known FAHs share a conserved N-terminal nucleotide binding motif for non-covalent attachment of the essential flavin adenine dinucleotide (FAD) cofactor despite their high overall sequence diversity (Wierenga et al. 1986; Kleiger and Eisenberg 2002). Therefore, all FAHs characterised so far are flavin-dependent proteins (Volkov et al. 2010; Joo et al. 2012a; Engleder et al. 2015; Hirata et al. 2015; Kang et al. 2017). Since the redox (reduction/oxidation) state of FAD does not change during substrate conversion, FAHs are belonging to the approx. 10% of flavoenzymes harbouring a non-redox active cofactor (Macheroux et al. 2011; Hemmi 2012). The most probable role of FAD in FAHs comprises the correct assembly of amino acids in the active site (Engleder et al. 2015). Additionally, a beneficial effect of FAD reduction to its two-electron reduced state was observed for OA hydration by OhyA, as well as the linoleic acid (LA) Δ9 hydratase from *Lactobacillus plantarum* AKU 1009a (CLA-HY) and OhyA1, but not for OhyA2 from *Stenotrophomonas maltophilia* (Takeuchi et al. 2014; Engleder et al. 2015; Kang et al. 2017). This is possibly due to facilitating protonation of the carbon-carbon double bond of the substrates (Macheroux et al. 2005; Engleder et al. 2015). This hypothesis coincides with the assumption that due to the in vivo redox milieu in the native bacterial hosts, reduced FAD rather than oxidised FAD might be associated with the oleate hydratase enzymes.

**Fig. 2:**
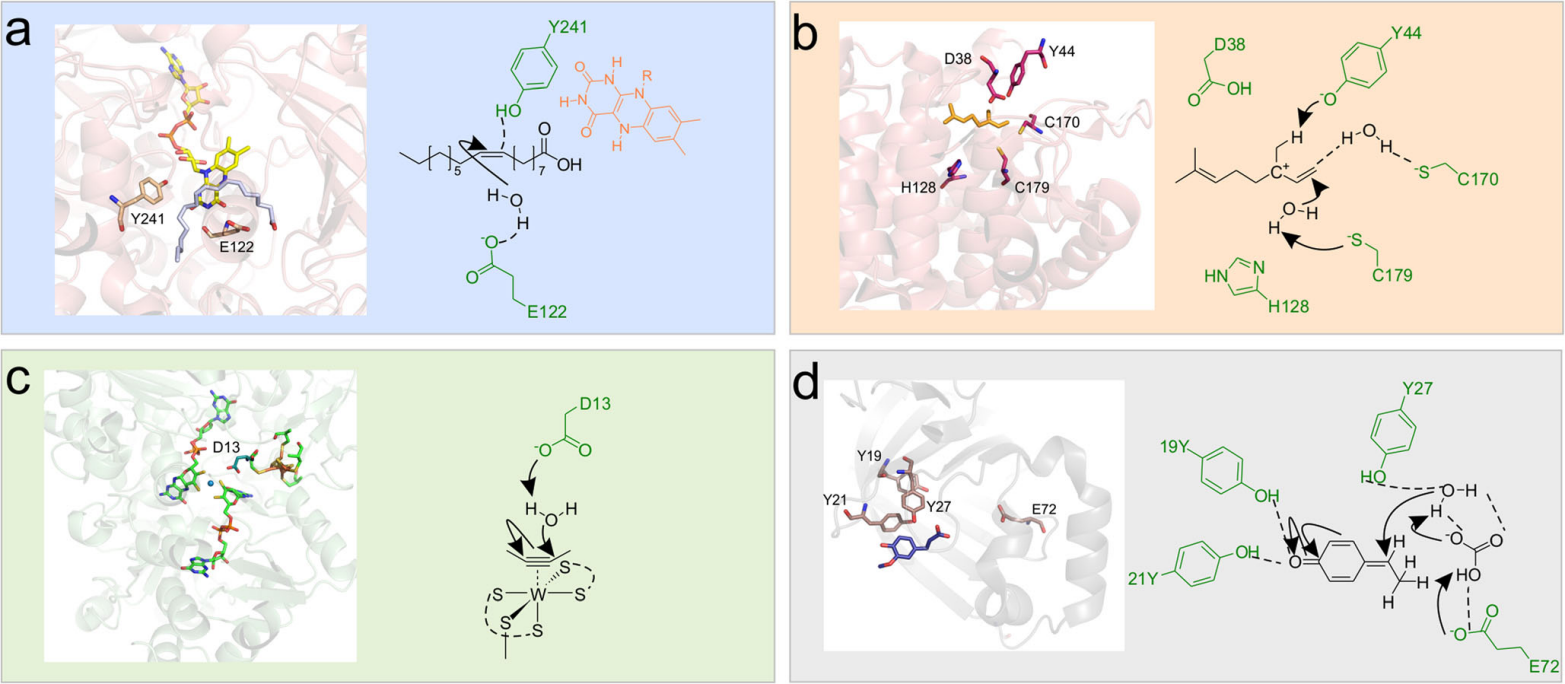
Active site architectures and proposed reaction mechanisms of hydratases catalysing water addition to non-activated carbon-carbon double and triple bonds. **a** Active site of *Elizabethkingia meningoseptica* oleate hydratase (OhyA; PDB-code: 4UIR) with docked oleic acid in grey, FAD in yellow and catalytic residues in light orange (Engleder et al. 2015). **b** Active site of *Castelaniella defragrans* linalool dehydratase-isomerase (LDI; PDB-code: 5HSS) with co-crystallised β*-*myrcene in orange and catalytic residues in magenta (Weidenweber et al. 2016). **c** Active site of *Pelobacter acetylenicus* acetylene hydratase (ACH; PDB-code: 2E7Z) showing both MGD cofactors and the tightly coordinated tungsten (blue), as well as the [4Fe:4S] cluster and the catalytic aspartate (Seiffert et al. 2007). (**d**) Active site of *Enterobacter* sp. ferulic acid decarboxylase (PAD; PDB-code: 3NX2) in complex with 3-(4-hydroxy-3-methoxyphenyl)-2-propenoic acid (dark blue) and residues important for substrate binding (light brown) and catalysis (Gu et al. 2011)

Recently, the amino acid sequences of 2046 putative FAHs were allocated to 11 homologous families (HFam1–11) in a hydratase engineering database (HyED) (Schmid et al. 2016). The authors identified a total of 80 conserved residues (present in > 90% of FAHs) among all sequences, many of which located either in the nucleotide binding motif or in the regions essential for catalysis and substrate binding. For other conserved positions, no function was proposed so far. Whereas database entries on new FAHs have been increasing rapidly, only relatively few 3D structures have been resolved to date. In fact, crystal structures of only four FAHs are available (Volkov et al. 2013; Engleder et al. 2015; Lorenzen et al. 2017; Park et al. 2018), and a homology model was built for a fifth enzyme (Ortega-Anaya and Hernández-Santoyo 2015). The first reported x-ray structure of a FAH was the structure of the LA hydratase from *Lactobacillus acidophilus* (LAH), classified in HFam2 according to the HyED (Volkov et al. 2013). In this work, structures without (apo-LAH) and with a bound substrate molecule (LA-LAH) were obtained, but co-crystallisation of the FAD cofactor was not successful. LAH adopts a homodimeric form comprised of four intricately connected domains, with domains I–III forming the main part accommodating both the substrate cavity and the putative cofactor binding site. The mainly α-helical fourth domain at the C-terminus was found to change its conformation upon binding of LA and was therefore suggested to form a lid covering the entrance to the hydrophobic substrate channel. A crystal structure of a FAH in complex with the essential FAD was first reported for OhyA (Engleder et al. 2015). The enzyme crystallised as a homodimer and is classified in HFam11. Similar to LAH, four domains were assigned, but in contrast to LAH, the non-covalently bound FAD co-crystallised in one of the subunits. Both monomers only differed notably in a loop region covering residues in the FAD binding pocket, which adopted a well-ordered conformation only upon presence of the cofactor. Identification of amino acid residues with roles in substrate binding to the V-shaped, hydrophobic binding channel and in catalysis led to the proposal of the first reaction mechanism for a FAH (Fig. 2a). Active site glutamate and tyrosine residues concomitantly catalyse the *anti*-addition of water to OA (Chen et al. 2015; Engleder et al. 2015). Despite their overall similarity, the 3D structures of LAH and OhyA displayed some notable variations in a loop region (L_98_–M_123_) covering the putative active site entrance, indicating conformational changes upon FAD binding and, possibly, a gating function of this region (Engleder et al. 2015). Both structures also comprise differently located binding sites and orientations of the substrate. The LA-LAH structure may reflect the initial recognition mode of a substrate at the surface of the protein, whereas substrate binding in the actual active site cavity appears to be depicted in the structure of OhyA (Volkov et al. 2013; Engleder et al. 2015; Ortega-Anaya and Hernández-Santoyo 2015).

In addition to the x-ray structures, a homology model based on ab initio and comparative modelling with the structural data of LAH was described for the *L. plantarum* CFQ-100 LA hydratase (LPH) (Ortega-Anaya and Hernández-Santoyo 2015). A homotrimeric form in solution was detected by gel filtration. Analyses of the modelled structure identified three domains and putative substrate binding sites near the surface and at the core of the molecule. Based thereon, a substrate recruiting mode similar to the one derived from structural data of LAH and OhyA was suggested. The third solved FAH structure was for the oleate hydratase from *Rhodococcus erythropolis* (OhyRe) from HFam3 (Lorenzen et al. 2017). OhyRe, which was not co-crystallising with FAD, is made up of four domains, but differs from the other structures by shorter N- and C-termini, as well as a monomeric state in solution. The different oligomerisation state was explained by oligomerisation in LAH and OhyA at the respective N- and C-terminal regions, which are missing in OhyRe. This caused the authors to speculate that all members of HFam3 class may be monomers. A sequence alignment of OhyRe with LAH and OhyA furthermore showed that the catalytically essential glutamate and a threonine with a proposed role in substrate binding in OhyA were replaced with a methionine and a valine in OhyRe. The activity of OhyRe variants M77E and V393T for hydration of OA was drastically reduced compared to the wild type enzyme, which suggested a different catalytic mechanism compared to the one reported for OhyA. Only recently, the 3D structure of dimeric *Stenotrophomonas* sp. KCTC 12322 oleate hydratase (OhySt, classified in HFam11) in its apo-form was reported (Park et al. 2018). In analogy to other hydratase structures, OhySt consists of four domains, in which only the fourth domain was structurally different from the previously described FAHs. Two loops close to the isoalloxazine and adenosine of FAD, respectively, adopted an entirely different conformation in OhySt than the equivalent regions in the structures of OhyA and LA-LAH. These extensive rearrangements upon binding of FAD to OhyA confirmed the structural role of flavin in active site arrangement among different FAHs. Collectively, the hitherto solved 3D structures of FAHs provided essential information on the mechanistic properties of this enzyme family. However, in order to unequivocally confirm substrate binding mode and catalytic mechanism, evaluation of a complete FAH structure in complex with cofactor and substrate(s) will still be required.

From a biotechnological viewpoint, FAHs offer a highly interesting route to functionalised fatty acids as compared to other enzymes described for these reactions, such as cytochrome P450 mono-oxygenases, lipoxygenases or epoxide hydrolases (Kim and Oh 2013; Kaprakkaden et al. 2017). Application of the latter enzymes is often limited by their low expression levels and poor stability, insufficient activity and the demand for stoichiometric amounts of nucleotide cofactors (Zorn et al. 2016). In contrast, FAHs are, in general, easily expressed in *Escherichia coli*, reasonably stable and active, and catalyse the reaction without the need for stoichiometric supply with FAD. Furthermore, FAHs allow for both excellent regio- and stereoselective lipid modification (Resch and Hanefeld 2015). Pioneering work on the stereoselectivity of FAHs was conducted in the 1960s (Schroepfer and Bloch 1963, 1965; Schroepfer 1966). In these studies, deuterated 10-HSA was produced microbially and subsequently dehydroxylated in an elegant series of organic reactions. By retention of this hydrogen after incubation of C9-deuterated stearic acid with *Corynebacterium diphtheriae*, which stereospecifically removed the *R*-hydrogen at C9 (Schroepfer and Bloch 1963), the authors deduced the absolute configuration of the hydroxyl group at C10 as the *R* enantiomer (Schroepfer 1966). Only in 2016, the original configurational assignment of 10-HSA was independently confirmed through comparison of the absolute configuration of (*S*)-10-HSA produced via asymmetric total synthesis with 10-HSA produced microbially (Brunner and Hintermann 2016). This work also provided a synthesis route for hydroxy fatty acids that should be general enough to produce reference material for stereochemical analysis of any long-chain n-hydroxy carboxylic acid.

Enzymatic hydration of fatty acids favours formation of the (*R*)-enantiomers, which is illustrated by determination of the stereochemical purity of products obtained either from microbial biotransformations or reactions with isolated enzymes. The enantiomeric excess (ee) of (*R*)-10-HSA obtained from microbial hydration by *Nocardia restrictus* ATCC 14887, *Mycobacterium fortuitum* UI-53387, *Pseudomonas* sp. NRRL-2994 and *Saccharomyces cerevisiae* was ranging from 82 to 98%, while *Nocardia aurantia* ATCC 12674 only produced enantiomeric mixtures of (*R*)- and (*S*)-10-HSA (Yang et al. 1993). For isolated FAHs, excellent ee values were reported in the case of OhyA (ee of 98% for (*R*)-10-HSA from OA) (Engleder et al. 2015), CLA-HY (ee > 99.9% for (*S*)-10-hydroxy fatty acids from LA, α- and γ-linolenic acid (LnA) (Takeuchi et al. 2014) and FA-HY1 from *L. acidophilus* NTV001 (ee > 99% for (*R*)-13-hydroxy fatty acids from LA and α-LnA) (Hirata et al. 2015). Contrary to the *S. cerevisiae*-mediated production of 10-HSA from OA (el-Sharkawy et al. 1992; Yang et al. 1993), more recent investigations are pointing towards hydration of OA by bacterial contaminants of employed yeast preparations (Brunner and Hintermann 2016; Serra and De Simeis 2017). Mixed bacterial culture isolates from commercial yeast samples gave rise to diastereomeric mixtures of 10-HSA, which suggested that the identification of distinct FAHs may lead to enzymes with unique enantioselectivity (Serra and De Simeis 2017). The regioselectivity as well as substrate and product profiles of FAHs have been reviewed recently (Hiseni et al. 2015). Due to the very recent discovery and functional characterization of additional enzymes, a revised list of all substrates converted by FAH activity, as well as the determined regioselectivities is given here (Table 1).

**Table 1:** Summary of FAHs tested for hydration of different free fatty acids

Organism enzyme		Myristoleic acid	Palmitoleic acid	Oleic acid	Ricinoleic acid	Linoleic acid	α-linolenic acid	γ-linolenic acid	Pinolenic acid	Stearidonic acid	References
	(Z)-9-undecenoic acid	(Z)-9-tetradecenoic acid	(Z)-9-hexadecenoic acid	(Z)-9-octadecenoic acid	(Z)-9–12-OH-octadecenoic acid	(Z)-9,12-octadecadienoic acid	(Z)-9,12,15-octadecatrienoic acid	(Z)-6,9,12-octadecatrienoic acid	(Z)-5,9,12-octadeca trienoic acid	(Z)-6,9,12,15-octadeca tetraenoic acid	
*Bifidobacterium animalis* subsp. *lactis* BB-12 *Ba*MCRA				10-OH		10-OH					(Yang et al. 2013)
*Bifidobacterium breve* NCFB 2258 *Bb*MCRA1				10-OH							(O’connell et al. 2013)
*Bifidobacterium breve* NCIMB 702258 *Bb*MCRA2			10-OH	10-OH		10-OH					(Rosberg-Cody et al. 2011)
*Chryseobacterium gleum* *Cg*OAH1	10-OH	10-OH	10-OH	10-OH		10-OH					(Schmid et al. 2016)
*Desulfomicrobium baculatum* *Db*OAH1	10-OH	10-OH	10-OH	10-OH		10-OH					(Schmid et al. 2016)
*Elizabethkingia meningoseptica* OhyA	10-OH	10-OH	10-OH	10-OH		10-OH	10-OH				(Bevers et al. 2009; Schmid et al. 2016)
*Gemella morbillorum* *Gm*OAH1	10-OH	10-OH	10-OH	10-OH		10-OH					(Schmid et al. 2016)
*Lactobacillus acidophilus* NTV001 FAHY1^a,b,c^			10-OH	10-OH		13-OH	13-OH	10-OH AND 13-OH	13-OH	13-OH	(Hirata et al. 2015)
*Lactobacillus acidophilus* NTV001 FAHY2				10-OH		10-OH	10-OH	10-OH			(Hirata et al. 2015)
*Lactobacillus acidophilus* LMG 11470 LHT10				10-OH		10-OH					(Kim et al. 2015)
*Lactobacillus acidophilus* LMG 11470 LHT13						13-OH	13-OH	13-OH	13-OH		(Kim et al. 2015)
*Lactobacillus acidophilus* NCFM *La-*LAH			10-OH	10-OH		10-OH	10-OH				(Yang et al. 2013; Volkov et al. 2013)
*Lactobacillus hammesii* DSM16381 *Lh*LAH						10-OH					(Chen et al. 2016)
*Lactobacillus plantarum* ST-III *Lp*MCRA				10-OH		10-OH					(Yang et al. 2013)
*Lactobacillus plantarum* AKU 1009a CLA-HY			10-OH	10-OH	10-OH	10-OH	10-OH	10-OH		10-OH	(Takeuchi et al. 2014)
*Lactobacillus plantarum* CFQ-100 LPH						10-OH					(Ortega-Anaya and Hernández-Santoyo 2015)
*Lactobacillus plantarum* TMW1.460 *Lp*LAH						10-OH					(Chen et al. 2016)
*Lactobacillus reuteri* LTH2548 *Lr*LAH						10-OH					(Chen et al. 2016)
*Lactobacillus rhamnosus* LGG *Lr*MCRA				10-OH		10-OH					(Yang et al. 2013)
*Lactobacillus spicheri* Lp38 *Ls*LAH						10-OH					(Chen et al. 2016)
*Lysinibacillus fusiformis* *Lf*OAH		10-OH	10-OH	10-OH	10-OH	10-OH	10-OH	10-OH			(Kim et al. 2012; Seo et al. 2013)
*Macrococcus caseolyticus* *Mc*OAH1		10-OH	10-OH	10-OH		10-OH AND 10,13-diOH	10-OH AND 10,13-diOH	10-OH AND 10,13-diOH			(Joo et al. 2012a)
*Rhodococcus erythropolis* OhyRe			10-OH	10-OH		10-OH	10-OH	10-OH			(Lorenzen et al. 2017)
*Stenotrophomonas sp.* KTCC 12332 OhySt				10-OH							(Park et al. 2018)
*Stenotrophomonas maltophilia* KCTC 1773 *Sm*OAH		10-OH	10-OH	10-OH		10-OH	10-OH	10-OH			(Joo et al. 2012b)
*Stenotrophomonas maltophilia* S028 OhyA1			10-OH	10-OH		10-OH	10-OH	10-OH			(Kang et al. 2017)
*Stenotrophomonas maltophilia* S028 OhyA2			10-OH	10-OH		10-OH	10-OH	10-OH			(Kang et al. 2017)
*Streptococcus pyogenes* M49 SPH			10-OH	10-OH		10-OH 10-OH and 13-OH	10-OH				(Volkov et al. 2010)

The regioselectivity of water addition is indicated by configuration of the hydrated carbon-carbon double bond(s) and the position(s) of the hydroxyl group(s) in the product. Only substrates with double bonds in cis-conformation are accepted and only enzymes characterized after isolation are listed

^a^Additional C18 fatty acids converted by FA-HY1: *cis*-vaccenic acid ((Z)-11-octadecenoic acid): 12-OH; aquilegic acid ((5E,9Z,12Z)-octadecatrienoic acid): 13-OH

^b^C20 fatty acids converted by FA-HY1: (Z)11,14-eicosadienoic acid: 15-OH; mead acid ((Z)-5,8,11-eicosatrienoic acid): 12-OH; (Z)5,11,14-eicosatrienoic acid: 15-OH; dihomo-γ-linolenic acid ((Z)-8,11,14)-eicosatrienoic acid): 12-OH and 15-OH; (Z)-11,14,17-eicosatrienoic acid: 12-OH and 15-OH; arachidonic acid ((Z)-5,8,11,14-eicosatetraenoic acid): 12-OH

^c^C22 fatty acid converted by FA-HY1: docosahexaenoic acid ((Z)-4,7,10,13,16,19-docosahexaenoic acid): 14-OH

In the majority of studies, enzymes were only tested for hydration of OA and LA, while shorter (C11 to C16) and longer (C20 to C22) substrates, as well as polyunsaturated fatty acids were employed much less frequently. In view of this non-standardized panel of test substrates and the varying reaction parameters among studies, a comprehensive comparison of the substrate specificity and activity of FAHs characterized to date is not possible. However, a strict regioselectivity for water addition to the *cis*-9 double bond of unsaturated fatty acids was observed for most enzymes, whereas FAHs hydrating the *cis*-12 double bond are underrepresented. In fact, since the discovery of a bacterial strain forming a 13-hydroxy fatty acid from LA (Hudson et al. 1998), only three enzymes catalysing the hydration of a *cis*-12 double bond were identified (Volkov et al. 2010; Joo et al. 2012a; Kim et al. 2015), and only the LA hydratase LHT-13 from *L. acidophilus* LMG 11470 added water exclusively to the *cis*-12 double bond of LA, α-LnA, and γ-LnA (Kim et al. 2015). A unique exemption from this apparently strict regioselectivity was discovered by functional characterization of FA-HY1 and FA-HY2 (57% amino acid sequence identity) from *L. acidophilus* NTV001 after heterologous expression in *E. coli* (Hirata et al. 2015). In keeping with most reports, highly regioselective hydration of only the *cis*-9 double bond was obtained by conversion of OA, LA, α-LnA, and γ-LnA with FA-HY2. In contrast, FA-HY1 hydrated *cis*-9, *cis*-11, *cis*-12, *cis*-13 and *cis*-14 double bonds in a total of 20 different mono-, di- and poly-unsaturated fatty acids with chain lengths between C16 and C22, making the relaxed substrate spectrum and broad regioselectivity of FA-HY1 unprecedented among all FAHs characterized so far.

Even though the currently available diversity of FAH permits selective lipid modification using various unsaturated fatty acids with different chain lengths and positions of the double bond(s), some prerequisites still appear mandatory for a substrate in order to be accessible for the hydration reaction. In essence, these can be summarized by five consensus requirements for FAHs (Hiseni et al. 2015; Demming et al. 2018):

1. A carbon-carbon double bond in *cis*-conformation
2. A free carboxylate of the fatty acid substrate
3. A chain length of at least C11 of an unsaturated fatty acid (Schmid et al. 2016)
4. A minimum distance of seven carbons between the carboxyl group and the hydrated *cis*-double bond
5. Addition of a terminal OH-group is not possible (Demming et al. 2017)

Through discovery of new FAHs and a more detailed characterization of known enzymes combined with inventive reaction engineering strategies, some formerly presumed restrictive properties of substrates for acceptance by a FAH were recently circumvented. The inherent limitations of at least seven carbons between the carboxyl group and the to be hydrated double bond were bypassed upon addition of short-chain saturated fatty acids as reaction additives (Marliere 2011; Atsumi et al. 2017; Demming et al. 2017). Using hexanoic acid as the co-substrate for hydration of 1-decene, approx. 50% of (*S*)-2-decanol were obtained after 4 days of incubating *E. coli* cells overexpressing OhyA (Demming et al. 2017). Similar approaches permitted (de)hydration of small alkenes (Marliere 2011) and water addition to ethylene upon addition of octanoic acid (Atsumi et al. 2017).

### Kievitone hydratase

Kievitone hydratase (KHS; EC 4.2.1.95) catalyses the formation of hydroxy-kievitone by addition of water to the prenylated isoflavanon kievitone. This conversion is inferred in the detoxification of the plant phytoalexin kievitone by *Fusarium* species upon infection of *Phaseolus vulgaris* (Kuhn and Smith 1979; Smith et al. 1982; Cleveland and Smith 1983). In addition to kievitone, fungal KHS activity was also induced by other plant flavonoids and isoflavonoids devoid of a 3-methylbut-2-en-1-yl moiety, such as phaseolin, biochanin A or rotenone, even though isolated KHS was not tested with these substrates (Turbek et al. 1990). Since the KHS reaction involves selective, cofactor independent formation of a tertiary alcohol, the enzyme offers intriguing potential for the production of important building blocks for the chemical industry (Jin and Hanefeld 2011). This is of particular relevance for the synthesis of (bio)polymers, pharmaceuticals and other fine chemicals, as currently available synthetic organic chemistry routes for tertiary alcohols are often limited by harsh reaction conditions and application of toxic reagents (Kourist and Bornscheuer 2011; Müller 2014). The biocatalytic potential of this reaction was further emphasized by the production of short-chain alkenes via KHS catalysed enzymatic dehydration of alcohols as described in a patent (Marliere 2009).

KHS was discovered in the late 1970s (Kuhn and Smith 1979) in culture filtrates of *Fusarium solani* f. sp. *phaseoli* (*Fs*KHS), and first characterized after partial isolation from fungal culture filtrates (Cleveland and Smith 1983). Concomitantly with activity for hydration of kievitone, *F. solani* culture filtrates also converted the isoflavonoid phaseollidin to hydroxy-phaseollidin with an additional enzyme distinctly responsible for phaseollidin hydration (Turbek et al. 1992). However, whereas *Fs*KHS has been subject to various studies, *F. solani* phaseollidin hydratase (EC 4.2.1.97) has not been further characterized so far.

Secretion of *Fs*KHS into the extracellular matrix is mediated by an N-terminal signal peptide (Turbek et al. 1990). *Fs*KHS is a homodimeric glycoprotein with good thermostability and maximum activity at slightly acidic pH and contains 6 conserved N-glycosylation sites (Cleveland and Smith 1983). Analysis of the complete *Fs*KHS nucleotide sequence allowed for identification of homologs in several other *Fusarium* species, which pointed to a ubiquitous presence of enzymes conferring KHS activity in this genus (Li et al. 1995). Indeed, a putative KHS nucleotide sequence with 58% sequence identity to *Fs*KHS was recently identified in *Nectria haematococca* MP VI on the basis of a similarity search (*Nh*KHS). The protein was expressed in secretory mode in *Pichia pastoris* and was purified form the culture supernatant. Functional characterization of *Nh*KHS confirmed its activity in formation of hydroxy-kievitone from kievitone, as well as biochemical properties similar to the ones obtained for *Fs*KHS. Furthermore, a role of N-glycosylation for activity rather than for overall stability was suggested (Engleder et al. 2018). Conversion of several other bioactive flavonoids (Karabin et al. 2014) in addition to kievitone revealed a relaxed substrate scope of *Nh*KHS. Most notably, hydration of the prenylated hops chalcone xanthohumol may provide facilitated access to hydroxy-xanthohumol, a natural compound with proven radical scavenging activity in human cancer cell lines in vitro (Tronina et al. 2013). Neither structural nor mechanistic studies on KHSs were described so far, but amino acid sequence alignments of different (putative) KHSs unveiled conserved regions clustered in the middle and C-terminal parts. Assuming that the degree of sequence conservation is an indicator for functional importance, these regions may be involved in either substrate binding or catalysis (Engleder et al. 2018).

### Carotenoid hydratase

Carotenoid 1,2-hydratases (EC 4.2.1.131) catalyse the hydroxyfunctionalisation of terminal carbon-carbon double bonds of carotenoids in various photosynthetic and non-photosynthetic bacteria (Scolnik et al. 1980; Armstrong et al. 1989; Kovács et al. 2003; Steiger et al. 2003; Giraud et al. 2004; Graham and Bryant 2009; Sun et al. 2009). A hydration mechanism for formation of hydroxycarotenoids was confirmed by incorporation of ^18^O-labelled water into neurosporene (Yeliseev and Kaplan 1997). The formation of the sterically demanding tertiary alcohol from carotenes usually increases the antioxidative effects of carotenoids compared to non-functionalized photosynthetic pigments (Albrecht et al. 1997, 2000; Sun et al. 2009).

So far, two evolutionarily distantly related groups of carotenoid 1,2-hydratases, classified either in the CrtC protein superfamily or in the CruF family, have been discovered (Sun et al. 2009). Carotenoid 1,2-hydratases are cofactor free enzymes with a molecular weight of approx. 35–45 kDa, and are associated with the plasma membrane in their natural hosts. Nevertheless, CrtCs from photosynthetic *Rubrivivax gelatinosus* and *Rhodobacter capsulatus* were obtained from the respective soluble fractions in active form and were used for comparative in vitro substrate scope studies (Steiger et al. 2003). *R. capsulatus* CrtC converted lycopene, neurosporene and the respective 1-hydroxycarotenoids, whereas the CrtC from *R. capsulatus* was not able to introduce a second hydroxyl group into carotenoid substrates. In 2011, *R. gelatinosus* and *Thiocapsa roseopersicina* CrtCs were biochemically characterized and challenged with different acyclic alkenes possessing a chain length between C5 and C20 (Hiseni et al. 2011). Some conversion was detected for the C20 substrate geranylgeraniol, but the inactivity on substrates shorter than C20 suggested a limitation of the substrate scope of CrtCs to only long-chain alkenes. In addition to enzymes converting exclusively acyclic substrates, the CrtC from *Thiodictyon* sp. was also active towards monocyclic carotenoids (Vogl and Bryant 2011), whereas the CrtC from *Chlorobium tepidum* efficiently hydrated monocyclic substrates, but was inactive on acyclic carotenoids (Frigaard et al. 2004). Collectively, these studies indicate that CrtCs from different organisms show substantially distinct substrate profiles.

Carotenoid 1,2-hydratases classified in the CruF family were discovered in the cyanobacterium *Synechococcus* sp. (Maresca et al. 2008; Graham and Bryant 2009). CruF orthologs are found in a wide range of carotenoid-synthesizing bacteria that do not contain a *crtC* gene, and are arranged in a separate phylogenetic clade with no overlaps to CrtCs (Graham and Bryant 2009; Sun et al. 2009). CruF catalyses the initial step in biosynthesis of the glycosylated carotenoid myxoxanthophyll in *Synechococcus* sp. and accepts both acyclic and monocyclic carotenoid substrates (Graham and Bryant 2009). The first CruF enzymes in non-photosynthetic bacteria were identified in two *Deinococcus* strains, but these only showed activity for the monocyclic carotenoid γ-carotene (Sun et al. 2009). As a detailed phylogenetic and functional comparison between CrtCs and CruFs has not been performed so far, it is currently unknown why and how two such distinct groups of carotenoid 1,2-hydratases may have evolved independently in different bacteria.

Even though structural data on CrtCs are not available, a putative mechanism for CrtC catalysed hydration of lycopene was proposed by Hiseni et al. (2016). From a comparative alignment of 100 CrtC-like amino acid sequences combined with a homology model and site-directed mutagenesis of conserved residues, they concluded that a highly acidic active site aspartate generates an intermediate carbocation at C2 of the substrate, which is then attacked by water to yield the tertiary alcohol. The suggested mechanism was analogous to the one reported for squalene-hopene cyclases, a class of terpene synthases that shares active site residues with conserved CrtC amino acids (Siedenburg and Jendrossek 2011). However, since the homology model was designed from a protein with only 17% sequence identity, the proposed reaction mechanism still needs to be validated with authentic structural data of a CrtC.

Only very recently, three other enzymes from the CrtC protein superfamily with conserved domain homology to carotenoid 1,2-hydratases were characterised. On the one hand, *Nh*KHS (Engleder et al. 2018) also possesses a characteristic CrtC domain and catalyses the addition of water to a non-activated carbon-carbon double bond as described in a separate chapter in this review. On the other hand, also PenF and AsqC, two enzymes catalysing unique epoxide rearrangements in fungal quinolone alkaloid biosynthesis feature the conserved CrtC domain, which may be important for providing a strongly acidic aspartate in these specific conversions (Zou et al. 2017).

### Linalool (de)hydratase-isomerase

Enzymatic water addition to monoterpenes provides access to high-value compounds from renewable, inexpensive starting material (Bicas et al. 2009). In this context, the bifunctional linalool dehydratase-isomerase (LDI, EC 4.2.1.127) is a unique enzyme that catalyses the reversible (de)hydration and isomerization of (*S*)-(+)-linalool, resulting in the formation of β-myrcene and geraniol, respectively (Brodkorb et al. 2010). In the hydration reaction, (*S*)-(+)-linalool can be generated from β-myrcene with high stereoselectivity (ee ≥ 95%) virtually without (*R*)-(−)-linalool formation (Lüddeke and Harder 2011). In view of its pleasant odour, (*S*)-(+)-linalool displays appealing properties for the cosmetics and fragrance industries. Since it is commercially hardly available, hydration of β-myrcene was suggested as an intriguing route for (*S*)-(+)-linalool production (Lüddeke and Harder 2011; Demming et al. 2018), even though its industrial application has not yet been reported. In contrast, LDI is already applied for the production of industrially highly relevant dienes such as isoprene and butadiene (Marliere 2013; Botes and Conradie 2015), which are amounting to market values of billions of dollars annually (Weidenweber et al. 2016).

Currently, the only known enzyme with sequence similarity to LDI is the membrane-bound linalool isomerase (LIS) from *Thauera linaloolentis* 47 Lol, with an overall amino acid identity of 20% (Marmulla et al. 2016). LIS and LDI share common properties regarding substrate affinity, temperature and pH optima, but LIS does not catalyse the (de)hydration reaction. Furthermore, an enzyme catalysing a similar reaction was discovered in *R. erythropolis* MLT1 (Thompson et al. 2010). Resting cells of this strain converted β-myrcene to geraniol, but little is known about the enzymatic system(s) mediating this reaction. The biotransformation was not completely inhibited by cytochrome P450 inhibitors, but product formation was quenched without oxygen supply. Clearly, the relevant enzymes for formation of geraniol from β-myrcene in *R. erythropolis* MLT1 remain elusive and require cloning for a detailed characterization of the pathway (Thompson et al. 2010).

LDI catalyses the initial steps of monoterpene mineralization in the facultatively anaerobic β-proteobacterium *Castellaniella defragrans* 65Phen when grown under anaerobic conditions with β-myrcene as the sole carbon source (Brodkorb et al. 2010). Since the thermodynamic equilibrium of the reactions favours isomerization of geraniol and dehydration of (*S*)-(+)-linalool, respectively, LDI may additionally confer detoxification of monoterpene alcohols in vivo. This was shown by 100- to even 1000-fold higher reaction rates for geraniol isomerization (*V*_max_ of approx. 25 μmol min^−1^ mg^−1^) and (*S*)-(+)-linalool dehydration (*V*_max_ of approx. 9 μmol min^−1^ mg^−1^) compared to the reverse reactions (*V*_max_ of approx. 8 nmol min^−1^ mg^−1^). LDI possesses an N-terminal signal sequence for SEC-dependent periplasmic translocation of the nascent polypeptide. The enzyme is not associated with a cofactor, but sensitive towards molecular oxygen and requires a mild reducing agent for full activity, suggesting that the reduction/oxidation state of its four cysteines are important for full activity (Brodkorb et al. 2010; Weidenweber et al. 2016).

Only recently, the crystal structure of *C. defragrans* LDI was independently solved by the groups of Harder and Hauer, respectively, either in complex with geraniol (Nestl et al. 2017) or with geraniol and β-myrcene (Weidenweber et al. 2016). In both instances, LDI crystallized as a cyclic homopentamer with a central hole, with each monomer showing an (α,α)_6_ barrel fold. While this fold is also observed in other proteins with similar functions (Wendt et al. 1997), the active site of LDI is located at the interface of two subunits, which is unprecedented among (α,α)_6_ barrel proteins. The importance of cysteines for LDI activity was established for an essential disulphide bond capping the substrate channel and for the contribution of the other two cysteines in the putative reaction mechanisms (Weidenweber et al. 2016; Demming et al. 2017). Both studies independently reported on acid/base catalysis for the (de)hydration and isomerization reactions of LDI, in which (*S*)-(+)-linalool is protonated by a cysteine, followed by rehydration for geraniol or deprotonation for β-myrcene (Fig. 2b). Nestl et al. furthermore proposed a mechanism implying the formation of a covalent thioterpene intermediate upon attack on the terminal alkene of (*S*)-(+)-linalool by an active site cysteine (Nestl et al. 2017). While both acid/base and covalent catalysis are plausible concepts for the LDI reactions, mechanistic studies will be needed to verify which catalytic mechanisms are tenable. Since a recent patent already demonstrated that LDI is a viable target for enzyme design (Marliere et al. 2016), mechanistic knowledge will be of major relevance for further development of structure-guided engineering towards unique diene and alcohol products.

Nestl et al. also provided the first detailed study on the substrate scope of LDI by challenging the enzyme with different linalool analogues and derivatives (Nestl et al. 2017). In all cases, the α-methylallyl alcohol signature motif was essential for dehydration. No activity was observed for linalyl amine and synthetic (*E*)-3,7-dimethylocta-1,4,6-trien-3-ol due to the higher nucleophilicity compared to water and the higher rigidity compared to accepted structures, respectively. Aside from that, 12 different substrates, including aromatic derivatives and ether analogs, as well as truncated and elongated structures were dehydrated with selectivity factors ranging from 5 to > 200 (Nestl et al. 2017).

### Limonene hydratase

One of the few enzymatic water addition reactions to a monoterpene in addition to the bifunctional LDI reaction is the regio- and stereoselective hydration of the 8,9-double bond of (*R*)-(+)-limonene to form α-terpineol by limonene hydratases (LIH) (Marmulla and Harder 2014). Due to its floral odour, (*R*)-(+)-α-terpineol is an essential raw material for the food and cosmetics industries. It is produced chemically at low costs by acid catalysed hydration and partial dehydration of pinene or crude turpentine oil (Rottava et al. 2010). However, in view of the increasing efforts industry is devoting to the production of natural flavours and fragrances, biotransformations routes towards enantiopure (*R*)-(+)-α-terpineol are highly desirable (Adams et al. 2003; Ran et al. 2008).

(*R*)-(+)-limonene is a bulk chemical accumulating as a major by-product during processing of citrus oil or wood to more than 50,000 t a^−1^ and therefore represents an attractive starting material for the biosynthesis of value-added flavour and fragrance compounds (Bicas et al. 2009). In general, α-terpineol production from limonene has been reported in a plethora of different bacteria and yeasts, including *Bacillus stearothermophilus*, *Pseudomonas gladioli*, *Sphingobium* sp., *Aspergillus* sp., *Fusarium oxysporum* and *Penicillium digitatum* (Duetz et al. 2003; Maróstica and Pastore 2006). However, considering that biotransformations of limonene were performed almost exclusively in whole cells with the co-production of many other limonene derivatives (Tan and Day 1998; Adams et al. 2003; Duetz et al. 2003; Kaspera et al. 2005; Rottava et al. 2010), specific enzymes for limonene hydration are almost never described. For instance, a hydration reaction was initially discussed for the highly selective production of (4*R*)-(+)-α-terpineol from (*R*)-(+)-limonene in *P. digitatum*, but later revised to a two-step reaction comprising an epoxidation and oxidative cleavage of the 8,9-double bond (Abraham et al. 1986; Tan et al. 1998; Pescheck et al. 2009; Badee et al. 2011). Furthermore, a thermostable LIH was implied in α-terpineol formation from limonene in recombinant *E. coli* expressing a limonene degradation pathway from *B. stearothermophilus*. Yet, only little information on the enzyme properties were given (Savithiry et al. 1997). Interestingly, the putative *B. stearothermophilus* LIH also hydrated the nitrile group of cyanopyridine.

In 1992, a membrane-associated LIH catalysing the stereoselective hydration of (4*R*)-(+)-limonene to (4*R*)-(+)-α-terpineol was isolated from *P. gladioli* and named α-terpineol dehydratase (Cadwallader et al. 1992). In addition to (4*R*)-(+)-limonene, the *S*-enantiomer was also converted to (4*S*)-(−)-α-terpineol, but the reaction rate was approx. 10-fold lower compared to the preferred stereoisomer (Cadwallader et al. 1992). Resting cells of *Sphingobium* sp. also converted (4*R*)-(+)- and (4*S*)-(−)-limonene to (4*R*)-(+)-α- and (4*S*)-(−)-α-terpineol, respectively, under both aerobic and anaerobic conditions without the need for cofactor supply (Bicas et al. 2010b). While production of (4*R*)-(+)-α-terpineol was highly stereoselective (ee ≥ 99%), the *S*-enantiomer was obtained with an ee of only 60%. The authors suggested conversion of both limonene enantiomers by a cofactor independent hydratase, but did not further confirm this assumption. Yet, so far, this is the highest level reported for (4*R*)-(+)-α-terpineol formation from (4*R*)-(+)-limonene at approx. 130 g L^−1^ of product after 96 h of biotransformation (Bicas et al. 2010b; Resch and Hanefeld 2015). Additionally, several studies on the oxygen-independent biotransformation of limonene by *F. oxysporum* indicated stereoselective formation of α-terpineol by a hydratase (Maróstica and Pastore 2006; Bicas et al. 2010a; Molina et al. 2015). Both limonene isomers were converted; but the activity for (4*R*)-(+)-limonene was 10-fold higher than for the *S*-enantiomer. In conclusion, access towards functionalized monoterpenoids with LIH appears to be a promising biocatalytic transformation, but some cases require additional investigations to confirm a hydration reaction for the production of α-terpineol from limonene.

### Acetylene hydratase

The anaerobic conversion of acetylene to acetaldehyde by acetylene hydratase (ACH, EC 4.2.1.112) is among the more peculiar enzyme-catalysed reactions characterised to date (Kisker et al. 1998; Meckenstock et al. 1999). Utilization of acetylenic compounds by bacteria was originally found about 60 years ago (Yamada and Jakoby 1959), and was characterized more thoroughly by the fermentative acetylene degradation in *Pelobacter acetylenicus* (Schink 1985; Meckenstock et al. 1999). So far, *P. acetylenicus* ACH is the only member of this enzyme group for which biochemical, structural and mechanistic properties of ACHs have been derived.

ACH is a monomeric protein (Rosner and Schink 1995), containing a [4Fe:4S] cluster and a molybdopterin guanine dinucleotide (MGD) cofactor-coordinated tungsten and is categorized in the DMSO reductase family of molybdenum and tungsten enzymes (Kisker et al. 1997). Currently, ACH is the only tungsten-dependent non-redox active enzyme known (Boll et al. 2016). The reaction optimum at 50 °C indicates a remarkable thermostability and its high sensitivity towards molecular oxygen emphasizes the importance of an anaerobic environment in the natural host (Rosner and Schink 1995). Elucidation (Einsle et al. 2005) and analysis (Seiffert et al. 2007) of the *P. acetylenicus* ACH structure identified four domains with structural similarity to other proteins of the DMSO reductase family. Yet, compared to other DMSO reductases, the region connecting domains II and III adopted an entirely different assembly. This caused exposure of a different portion of the metal coordination sphere towards the active site and helped to rationalize the differences between the reactions catalysed by ACH and other DMSO reductases. In the active site, the tungsten was tightly coordinated to the MGD cofactors, a water molecule and an active site aspartate (Fig. 2c). Docking of acetylene indicated that the substrate was fitting perfectly into the hydrophobic pocket (Seiffert et al. 2007).

Heterologous expression of *P. acetylenicus* ACH in *E. coli* Rosetta (DE3) only yielded reasonably active enzyme after N-terminal fusion of *E. coli* chaperone NarG, allowing for adequate incorporation of the tungsten cofactor into the unfolded polypeptide chain (TenBrink et al. 2011). Site-directed mutagenesis of the active site aspartate to alanine and glutamate resulted in almost complete inactivation of the enzyme in the first case, whereas the latter exchange did not have any notable effect on activity. Similarly, an ACH variant harbouring a mutation of isoleucine to alanine in the hydrophobic binding pocket showed a marked loss of activity. These results supported earlier evidence that the carboxylate of the active site aspartate was supposedly crucial for the catalytic mechanism and that the environment of the binding pocket was specifically adjusted for accommodation of the substrate. This was consistent with substrate specificity studies, in which ethylene, cyanide, nitriles, isonitriles and acetylene derivatives were not converted (TenBrink et al. 2011).

Despite the availability of detailed biochemical and structural data, the reaction mechanism of ACHs is still disputed (Boll et al. 2016). Amid their structural investigations, Seiffert et al. suggested hydration via electrophilic attack on the substrate by tungsten-bound water in a Markovnikov-type addition as the most probable mechanism (Seiffert et al. 2007). However, since more recent studies showed that this would be obstructed by high-energy barriers, an alternate mechanism supported by quantum chemical calculations was proposed (Liao et al. 2010). Therein, water was first displaced from tungsten by the substrate, followed by deprotonation of this water by the active site aspartate. Subsequently, activated water would perform a nucleophilic attack on the substrate, forming vinyl anion and vinyl alcohol intermediates, before spontaneously tautomerising to acetaldehyde (Fig. 2c). While this mechanism still awaits experimental confirmation, e.g. by determining the real protonation state of the active site aspartate (Boll et al. 2016), it provided a conclusive mechanistic explanation for the observed chemoselectivity of the ACH reaction (Liao and Himo 2011).

### Promiscuous hydratase activity of decarboxylases

Despite all the recent advances, the broad application of hydratases in organic synthesis is undermined by their poor flexibility, ultimately leading to a narrow substrate tolerance. Discovery of hydration biocatalysts with a more relaxed substrate scope, or even of enzymes showing promiscuity for water addition are therefore long-standing aims in industrial biotechnology (Hult and Berglund 2007; Turner 2009).

Such promiscuous hydratase activity was for the first time reported for phenolic acid decarboxylases (EC 4.1.1.102) from *L. plantarum* (PAD_*Lp*) and *Bacillus amyloliquefaciens* (PAD_*Ba*), which formally catalysed the hydration of *p*-vinylphenol in addition to the natural decarboxylation reaction (Wuensch et al. 2013). An enzyme screening revealed that several bacterial PADs and ferulic acid decarboxylases (FDCs) were able to catalyse the *S*-selective hydration of different *p*-vinylphenol derivatives with ee values ranging from 3 to 53% with only little background carboxylation. Hydration was strongly dependent on the concentration of bicarbonate in the reaction buffer, which caused the authors to suppose its participation in the catalytic mechanism by permitting the formation of a quinine methide intermediate (Wuensch et al. 2013). A somewhat different picture was deduced from quantum mechanical calculations based on the PAD from *Bacillus subtilis*, which indicated that the energy barrier for formation of the substrate-bicarbonate intermediate might be too high (Sheng and Himo 2017). Instead, it was proposed that the quinine methide is formed by protonation of the double bond of *p*-vinylphenol, followed by nucleophilic attack of water and release of the product (Fig. 2d). The bicarbonate may assist this process by providing a concerted proton shuttle between an acidic glutamate, the substrate and water, rationalizing the enhancing—but not essential—role of bicarbonate in the reaction (Wuensch et al. 2013; Sheng and Himo 2017).

The versatility of PAD-catalysed hydration was recently extended to the addition of several C-, N- and S-nucleophiles to *p*-vinylphenol (Payer et al. 2017). Six out of 17 tested non-natural nucleophiles were accepted by different bacterial PADs and FDCs with moderate to good conversion and ee values for the *S*-enantiomer, respectively. Mechanistic investigations were performed in analogy to the study discussed above, from which a similar mechanism for nucleophile addition was derived (Payer et al. 2017; Sheng and Himo 2017).

## Conclusion and future perspective

The asymmetric hydroxyfunctionalisation of alkenes is one of the toughest challenges in modern organic synthesis (Kourist and Bornscheuer 2011; Jin and Hanefeld 2011; Müller 2014). In nature, this objective is addressed by direct addition of water to carbon-carbon double bonds with hydratases in a highly selective reaction with 100% atom economy. While hydratases have been known for almost 100 years, their value for industrial biocatalysis was not envisioned until recently, and their full potential has not nearly been exploited, yet. Only during the last 15 years, research on hydratases in both academia and industry has intensified, resulting in the identification and characterization of new hydratases, as well as in a remarkable increase on the biochemical and structural information on top of previously described enzymes. Since the first crystal structure was solved in 2005, various additional 3D structures of hydratases were determined. They allowed for insight into active site architectures and strongly suggested that addition of water to non-activated carbon-carbon double bonds is generally conferred by acid/base catalysis using charged amino acid side chains for activation of substrate and water. The increasingly more detailed characterization of metabolic pathways revealed involvement of (putative) hydration reactions in biosynthetic routes for valuable secondary metabolites, and the constant progress in gene sequencing methods permitted discovery of new hydratases from established or newly designed databases. Finally, identification of promiscuous hydration activity in well-known enzymes expanded the portfolio of hydration biocatalysts beyond inferred limitations.

While thorough substrate specificity studies showed that the supposedly limited substrate spectrum of hydratases does actually not hold true in some cases, this limitation is still considered the major challenge for hydration biocatalysis in the near future. In order to surmount this hurdle, the systematic development of hydratases for biocatalytic applications may be grouped into two approaches. On the one hand, detailed mechanistic and structural studies of active site architectures will lead to a better understanding and, ultimately, to novel rational protein design strategies for specific applications. On the other hand, comparative structure and sequence analyses with powerful bioinformatics’ tools and utilization of the increasing quantity of (meta)genomics’ data from public databases will lead to the discovery of new hydratases from unexplored natural diversity (Bornscheuer 2018). Taking these concepts as the basis for future efforts will contribute to developing tailor-made hydration biocatalysts for the next generation of industrial biotechnology.
